# The Development of the Older Persons and Informal Caregivers Survey Minimum DataSet (TOPICS-MDS): A Large-Scale Data Sharing Initiative

**DOI:** 10.1371/journal.pone.0081673

**Published:** 2013-12-04

**Authors:** Jennifer E. Lutomski, Maria A. E. Baars, Bianca W. M. Schalk, Han Boter, Bianca M. Buurman, Wendy P. J. den Elzen, Aaltje P. D. Jansen, Gertrudis I. J. M. Kempen, Bas Steunenberg, Ewout W. Steyerberg, Marcel G. M. Olde Rikkert, René J. F. Melis, on behalf of TOPICS-MDS Consortium

**Affiliations:** 1 Department of Geriatric Medicine, Radboud University Medical Center, Nijmegen, Netherlands; 2 Anu Research Centre, University College Cork, Cork, Ireland; 3 Department of Epidemiology, University of Groningen, University Medical Center, Groningen, Netherlands; 4 Department of Internal Medicine and Geriatrics, Academic Medical Center, Amsterdam, Netherlands; 5 Department of Public Health and Primary Care, Leiden University Medical Center, Leiden, Netherlands; 6 Department of General Practice and Elderly Care Medicine/EMGO + Institute for Health and Care Research, VU University Medical Center, Amsterdam, Netherlands; 7 CAPHRI School for Public Health and Primary Care, Department of Health Services Research, Maastricht University, Maastricht, Netherlands; 8 Julius Center for Health Sciences and Primary Care, UMC Utrecht, Utrecht, Netherlands; 9 Department of Public Health, Erasmus MC University Medical Center, Rotterdam, Netherlands; Cardiff University, United Kingdom

## Abstract

**Introduction:**

In 2008, the Ministry of Health, Welfare and Sport commissioned the National Care for the Elderly Programme. While numerous research projects in older persons’ health care were to be conducted under this national agenda, the Programme further advocated the development of The Older Persons and Informal Caregivers Survey Minimum DataSet (TOPICS-MDS) which would be integrated into all funded research protocols. In this context, we describe TOPICS data sharing initiative (www.topics-mds.eu).

**Materials and Methods:**

A working group drafted TOPICS-MDS prototype, which was subsequently approved by a multidisciplinary panel. Using instruments validated for older populations, information was collected on demographics, morbidity, quality of life, functional limitations, mental health, social functioning and health service utilisation. For informal caregivers, information was collected on demographics, hours of informal care and quality of life (including subjective care-related burden).

**Results:**

Between 2010 and 2013, a total of 41 research projects contributed data to TOPICS-MDS, resulting in preliminary data available for 32,310 older persons and 3,940 informal caregivers. The majority of studies sampled were from primary care settings and inclusion criteria differed across studies.

**Discussion:**

TOPICS-MDS is a public data repository which contains essential data to better understand health challenges experienced by older persons and informal caregivers. Such findings are relevant for countries where increasing health-related expenditure has necessitated the evaluation of contemporary health care delivery. Although open sharing of data can be difficult to achieve in practice, proactively addressing issues of data protection, conflicting data analysis requests and funding limitations during TOPICS-MDS developmental phase has fostered a data sharing culture. To date, TOPICS-MDS has been successfully incorporated into 41 research projects, thus supporting the feasibility of constructing a large (>30,000 observations), standardised dataset pooled from various study protocols with different sampling frameworks. This unique implementation strategy improves efficiency and facilitates individual-level data meta-analysis.

## Introduction

Demographic shifts towards an older population have given rise to new health care challenges across high-income countries. Despite general improvements in self-perceived health over time, health expectancy metrics have revealed increases in life years with chronic co-morbidity and mild functional impairment[1]. As health profiles of populations change, contemporary health care systems must be re-evaluated to ensure the best provision of care to older persons with more complex needs. 

Such is the case for the Netherlands, where an estimated 10% of the population will be 85 years or older by the year 2050[2]. With the aim of developing a more proactive, integrated health care system to accommodate the growing number of older patients, in 2008, the Dutch Ministry of Health, Welfare and Sport commissioned the National Care for the Elderly Programme. Under this Programme, a network of local health care providers, consumer advocates and research centres was established with the guiding principles of improving care, quality of life and self-reliance among older persons. To achieve these goals, the Programme promoted research in physical, mental and social health and well-being. 

While numerous research projects were to be conducted under this national agenda, the Programme further advocated the development of The Older Persons and Informal Caregivers Survey Minimal DataSet (TOPICS-MDS) which would be integrated into all funded research protocols. This framework would not only have the intrinsic advantage of gathering uniform information on a large sample of older persons and caregivers at minimal cost but also promote data sharing between institutions. The Programme envisioned individual patient data could then be pooled to facilitate meta-analysis as well as serve as a public repository for external users. 

Internationally, policymakers, geriatricians and other health professionals have long recognised the utility of incorporating minimal data collection as part of routine management in care facilities[3,4] and hospitals[5] as a well as a mechanism to achieve standardised outcome measurements in research[6,7]. In this context, TOPICS-MDS was developed to serve as a complementary instrument which would not only collect information on older persons but also informal caregivers and health services utilisation. TOPICS-MDS therefore has a broader scope than previous minimal datasets on older persons’ health and contains data relevant for many disciplines, including gerontology, public health and health economics. 

Given that TOPICS-MDS was created as a large-scale data sharing initiative[8], the aim of this first paper on the database was two-fold: (1) to describe the development of TOPICS-MDS and feasibility of data collection; and (2) to discuss how frequently met challenges in building a public data repository were overcome.

## Materials and Methods

### Project management and governance

TOPICS-MDS project was carried out as a collaborative effort between the eight medical research centres in the Netherlands, with Radboud University Medical Center serving as the central institution. A Project Group was established to advise on the development and maintenance of TOPICS-MDS and comprised of twelve members, a single representative from each medical centre and four additional working group members with expertise in database management and epidemiology. To ensure the commitment of all involved parties, TOPICS-MDS project was overseen by a nationally representative Steering Committee comprised of eight stakeholders from different geographical regions within the national network.

### Development of the minimal dataset instrument

Since TOPICS-MDS instrument would be incorporated into a range of research projects, it was therefore critical that the instrument was finalised prior to the commencement of these projects. Thus, the first priority of the National Care for the Elderly Programme was to develop a concise, standardised instrument which would collect essential information on the health status of the older persons and informal caregivers. Using validated instruments for use in older populations, a small working group was nominated to draft a prototype for TOPICS-MDS instrument. The working group outlined key domains and data points for the initial prototype. Several revisions of TOPICS-MDS instrument were undertaken before consensus was achieved among working group members. Upon consensus, an independent multi-disciplinary panel with expertise in gerontology, epidemiology, biostatistics and health services research was invited to evaluate the instrument’s content and utility. Only minor revisions were warranted from the panel’s feedback. 

TOPICS-MDS was then piloted in four regions throughout the Netherlands. A descriptive analysis was conducted to identify patterns in missingness. Two main operational issues were observed; several questions were consistently misinterpreted due to either (1) linguistic construct or (2) lay-out. Thus, a plain language expert was commissioned to revise TOPICS-MDS instrument for clarity and readability, and a finalised version of the instrument was approved. The English translations of the surveys administered to older persons and informal caregivers are available at: http://www.topics-mds.eu.

### Included measurements: Older persons

For older persons, information was collected on demographics, morbidity, quality of life, functional limitations, mental health, social functioning and health service utilisation for a total of 51 data points. 

#### Demographics

The following demographic characteristics were included in TOPICS-MDS: sex; age; marital status; country of origin; primary domicile (e.g. independent residence, retirement home, nursing home); educational level and socio-economic status. In accordance with the Dutch educational system, educational level was classified into seven categories, with the lowest category representing less than primary school and the highest representing college/postgraduate education. Socio-economic status was categorised according to the Dutch Social and Cultural Planning Office Socio-Economic Status Index[9]. For this index, respondents’ residential post codes were linked to geospatial data on average income, employment type and educational level to create an overall summary score, with higher numbers indicating higher socioeconomic status.

#### Morbidity

Respondents were asked to indicate morbidities experienced in the last twelve months from 17 pre-defined conditions (e.g. diabetes, asthma, cancer). Included conditions were based on a listing widely used in the Netherlands to record multimorbidity[10]. The presence of two or more conditions from this listing indicates multi-morbidity. 

#### Quality of life

The EuroQol Five Dimensional scale (EQ-5D)[11] is recognised as an optimal instrument to derive preference based quality of life values, particularly when brevity is required[12]. For the purposes of this project, a modified version of the EQ-5D, the EQ-5D+C, was used[13]. Whereas the traditional EQ-5D assesses five attributes (mobility, self-care, usual activities, pain/discomfort, anxiety/depression), the EQ-5D+C includes an additional attribute to assess cognitive function. Each attribute has three response options (‘no problems’, ‘some problems’ or ‘extreme problems’), resulting in a score or ‘1’, ‘2’ or ‘3’ respectively. Individual attribute scores are then concatenated into a six-digit number to describe a respondent’s health state, with ‘111111’ representing the best possible health state and ‘333333’ the worst possible health state. Thus, this metric has the potential to describe up to 729 (3^6^) unique health states. To date, there is no validated weighting formula to convert the EQ-5D+C health state to a summary index in the Dutch population[13]. However, such weightings are available for the EQ-5D[14], and EQ-5D summary scores are available in TOPICS-MDS. 

Respondents were also asked to rate their current quality of life from a five-level response option ranging from ‘poor’ to ‘excellent’ and their quality of life relative to the previous year from a five-level response option ranging from ‘much worse’ to ‘much better’. These two questions were formed using phrasing similar to self-perceived health questions from the RAND-36, which is an internationally recognised health-related quality of life survey validated for use in the Netherlands[15,16]. Self-perceived quality of life was further assessed with a modified version of Cantril’s Self Anchoring Ladder[17], where respondents were asked to rate their present life on a scale between zero and ten.

#### Functional limitations

The extent of functional limitations was measured using a modified version of the Katz Index of Independence Basic Activities of Daily Living (ADL), Instrumental Activities of Daily Living (IADL) and an additional indicator of mobility[18]. To measure functional limitations, respondents were asked if assistance is required for six basic functions (i.e. bathing; dressing; eating; toileting; use of incontinence products; getting up from a chair) and seven instrumental functions (i.e. grooming; use of telephone; travelling; grocery shopping; meal preparation, household tasks; taking medications; financial management). To measure mobility, respondents were asked if assistance was required while walking. Metrics to assess ADL and IADL, such as the Katz Index, have been administered in a variety of geriatric populations[19] and has been shown to produce reliable results irrespective of completion by a respondent or a proxy[18,20]. Responses are rated on a binary scoring system (dependent=1; independent=0) and summated, with higher scores representing greater functional limitations. 

#### Emotional wellbeing

The Rand-36 mental health sub-scale[16] reliably measures a unidimensional concept of mental state[21], and was therefore utilised to assess psychological wellbeing in TOPICS-MDS. The sub-scale is comprised of five questions asking respondents how often in the past four weeks they have felt (1) very nervous, (2) calm and peaceful, (3) down-hearted and blue, (4) happy and (5) so down in the dumps nothing could cheer [them] up. Five-level mutually exclusive response options are available ranging from ‘never’ to ‘always’. Positive attributes (e.g. feeling happy) are scored from zero to 100 respectively, whereas negative attributes (e.g. feeling very nervous) are reverse scored. Individual item scores are averaged and rescaled to produce a summary score between zero and 100, with higher scores indicating a more positive emotional state.

#### Social functioning

Social functioning was determined by a single question derived from the RAND-36[16]. Based on a five-level response option (from ‘never’ to ‘regularly’), respondents were asked how often in the past four weeks their physical health or emotional problems had hampered their social activities.

#### Health services utilisation

The number of hospital admissions, length of hospital stay and urgent care visits occurring in the twelve-month prior to administration of the survey were collected. Information on the frequency of home care assistance (e.g. community nurse) and temporary residence in a care home or a nursing home were also recorded.

### Included measurements: Informal caregiver

For the informal caregiver, information was collected on demographics, hours of informal care and quality of life for a total of 27 data points.

#### Demographics

The following demographic characteristics were included in TOPICS-MDS: sex; age; socioeconomic status, the caregiver’s relationship with the care recipient; whether the caregiver resided with the care recipient, and if not, the geographical distance between the caregiver and care recipient.

#### Hours of informal care

Respondents were asked to retrospectively indicate how many hours in the past week they assisted with household tasks, personal care, transport or financial/administrative duties. Notably, despite the potential for recall bias, retrospective reports of hours of informal care can yield valid and reliable results in cross-sectional studies if adjustments for multi-tasking are included in the analysis[22].

#### Quality of Life

Similarly to older persons, self-perceived quality of life was measured using two questions adapted from the RAND-36[16] and the modified version of Cantril’s Self Anchoring Ladder[17]. However, given that a high level of burden among a caregiver can negatively impact the physical and mental wellbeing of both the caregiver and care recipient[23], subjective care-related burden was measured using the validated CarerQol-7D[24]. The CarerQol-7D was modelled after the EuroQol 5-D and includes seven attributes: care-related fulfilment; relational problems with the care recipient; mental health; time management; financial security; social support; and physical health. From three response options (‘no’, ‘some’, ‘a lot’, scored as ‘1’, ‘2’ and ‘3’ respectively), respondents can indicate the extent of each attribute in their personal situation[24]. The CarerQol-7D score is derived likewise to the EuroQol 5-D+C[13] and thus can describe up to 2,187 (3^7^) levels of care-related burden. 

Care-related burden was further assessed with the CarerQol-VAS[24], which uses a visual analogue scale ranging from ‘0’ to ‘10’ to rate a caregiver’s level of happiness from 'completely unhappy' to 'completely happy'. Difficulty of care provision and the level happiness if another were to assume care responsibilities were also rated with a VAS.

### Sampling framework and longitudinal data collection

In total, 52 independent research studies included TOPICS-MDS into their research protocols. The study design, sampling framework and inclusion criteria differed across research studies. Several individual project protocols included longitudinal data collection. In such cases, TOPICS-MDS instrument was administered at baseline and at least one additional follow-up was scheduled, typically 12 months after baseline.

### Ethical approval

TOPICS-MDS instrument was integrated into pre-existing research protocols, and therefore ethical approval for the collection of TOPICS-MDS was sought from individual study sites. Results presented in this analysis were exempt from institutional review as data were anonymised and within the public domain.

### Data collection and management

A data dictionary and a standardised protocol for data cleaning procedures were drafted and provided to all participating project managers. To preserve participant confidentiality, data were cleaned at individual research sites, stripped of any personal identifiers and entered into a standardised computerised database. All data were submitted to a centralised body (Radboud University Medical Center) for the collation of a national dataset.

### Development of a public data repository

To facilitate external users, all de-identified data maintained in the public repository have been verified for accuracy and clearly labelled. A single institution (Radboud University Medical Center) was nominated to be the custodian of TOPICS-MDS and facilitate incoming data requests. To ensure equitable use, the Project Group drafted a data access policy and selected a Societal Board to review the societal merits and benefits of all data requests. Members of the Societal Board were nominated by the National Care for the Elderly network and comprised of seven members: a consumer advocate (chair), two research scientists, two community representatives and two health policy professionals. 

Following data collection, there is a six-month moratorium in the release of the data. After this period, TOPICS-MDS data access policy permits all research scientists affiliated with an academic, healthcare or other research institution worldwide to submit a request to access data. Prior to the release of data, all requests must be approved by both the Project Group and Societal Board.

## Results

TOPICS-MDS contains essential data to better understand health challenges experienced by older persons and informal caregivers. To highlight the utility of the TOPICS-MDS, descriptive statistics were calculated using preliminary data from 41 research projects. Results are shown for select characteristics only. All analyses were performed using SAS 9.2 (Carey, NC, USA)

Between 2010 and 2013, a total of 41 research projects contributed data to TOPICS-MDS. The majority of studies sampled were from primary care settings and inclusion criteria differed across studies (Table 1). The following analyses are based on the preliminary data for 32,310 older persons and 3,940 informal caregivers.

**Table 1:** Characteristics of 41 projects included in The Older Persons and Informal Caregivers Minimal DataSet, Netherlands, 2012.

	**Projects**	**Participants**
	**(N=41)**	**(N=32,310)**
Sampling frame	N	N (%)
Primary care practice	20	16,537 (51.2)
General population	10	6,401(19.8)
Hospital	5	1,753 (5.4)
Retirement community	3	5,083 (15.7)
Nursing home	3	2,536 (7.9)
Inclusion criteria^a^		
Frailty^b^	6	8,832 (27.3)
Dementia	3	2,352 (7.3)
Age minimum		
45 years	1	1,479 (4.6)
50 years	1	535 (1.7)
60 years	4	1,661 (5.1)
65 years	14	8,800 (27.2)
70 years	6	1,688 (5.2)
75 years	6	10,876 (33.7)
Unspecified	9	7,271 (22.5)

^a^ Inclusion criteria presented in this table are neither exhaustive nor mutually exclusive.

^b^ Operational definitions for frailty differed across studies.

The majority of older persons in this cohort were women (59.0%). Relative to men, women were modestly older, more likely to be widowed and more likely to reside independently (Table 2). Multi-morbidity was common, with three-quarters of respondents reporting two or more morbidities. The most frequently cited conditions were hearing problems (45.8%), knee and hip joint damage (42.0%) and vision disorders (39.4%). 

**Table 2:** Demographic characteristics of older persons, The Older Persons and Informal Caregivers Minimal DataSet, Netherlands, 2012.

	**Men**	**Women**
	**(N=13,237)**	**(N=19,017)**
Age (mean, SD)	77 (8)	79 (8)
Marital status		
Married	70.7	35.8
Widowed	18.3	50.4
Other^a^	11.1	13.8
Dutch origin	91.5	91.1
Primary domicile		
Independent residence	27.5	50.7
Residence with family members	51.2	29.7
Retirement home	19.9	17.0
Nursing home	1.4	2.5
Educational level^b^		
Primary school or less	25.6	42.2
Practical/secondary vocational training	47.3	44.9
Some college/university degree	27.2	12.9

Note: Values are presented as percentage unless otherwise stated. Data are based on 41 research projects.

^a^ Includes single, divorced and cohabiting.

^b^ Collapsed from seven Dutch educational categories.

Based on the EQ-5D+C, the vast majority reported no problems with self care; though, severe problems with anxiety and depression were relatively high among both men and women (Table 3). One-third (32.9%) of respondents reported no functional limitations in ADL or IADL. However, substantial gender disparities were observed, with women less likely to report no functional limitations than men (25.2% versus 47.5% respectively). The prevalence of incontinence products use and requiring assistance with household tasks and walking were notably higher among women (Table 4).

**Table 3:** Percentage of older persons reporting no, some or severe problems on the EQ-5D+C quality of life scale by sex, The Older Persons and Informal Caregivers Minimal DataSet, Netherlands, 2012.

	**No problem**	**Some problems**	**Severe problems**
Mobility			
Men	50.6	47.9	1.5
Women	37.8	60.3	1.9
Self-care			
Men	83.3	12.4	4.3
Women	77.2	16.5	6.2
Usual activities			
Men	63.4	26.5	10.1
Women	53.0	36.4	10.6
Pain/discomfort			
Men	48.7	45.6	2.3
Women	33.9	55.4	10.9
Anxiety/depression			
Men	71.0	18.3	10.7
Women	62.2	25.1	12.7
Cognitive functioning			
Men	64.9	32.9	2.2
Women	67.9	30.2	2.0

Note: Percentages are based on 13,237 men and 19,017 women.

**Table 4:** Percentage of older persons requiring assistance for activities of daily living by sex, The Older Persons and Informal Caregivers Minimal DataSet, Netherlands, 2012.

	**Men**	**Women**
	**(N=13,237)**	**(N=19,017)**
**Basic activities**		
Bathing or showering	14.6	21.3
Dressing	10.5	13.6
Eating	3.3	3.1
Toileting	5.2	5.6
Use of incontinence products	11.9	39.8
Getting up from a chair	7.2	10.1
**Instrumental activities**		
Grooming	6.0	6.1
Use of telephone	8.7	6.4
Travelling	23.8	40.8
Grocery shopping	20.2	36.5
Preparing a meal	26.5	23.3
Household tasks	39.6	62.5
Taking medication	13.1	12.5
Financial management	16.1	20.8
**Mobility**		
Walking	18.9	31.9

The average age of informal caregivers in this cohort was 63 years (SD 13); more than two-thirds (69.8%) were women. Caregivers were most likely to be a spouse/life partner or a daughter/son (in-law) of the care recipient (42.5% and 40.2% respectively); more than half (52.3%) did not live with their care recipient. Approximately 10% of caregivers reported ‘some’ or ‘a lot’ of care-related financial burden and nearly one-third (30.6%) received no social support from family, friends or acquaintances (Table 5). 

**Table 5:** Percentage of caregivers reporting problems on the CarerQol-7D quality of life scale (n=3,940), The Older Persons and Informal Caregivers Minimal DataSet, Netherlands, 2012.

	**No**	**Some**	**A lot**
Satisfaction performing care tasks	5.9	41.0	53.1
Relational problems with care recipient	52.6	34.2	13.3
Issues with personal mental health	49.9	37.1	12.9
Issues with personal physical health	44.3	40.5	15.2
Problems combining daily activities and care tasks	49.6	38.3	12.1
Financial problems	90.7	7.3	2.0
Social support in care tasks	30.6	39.0	30.5

## Discussion

TOPICS-MDS has been successfully incorporated into numerous research projects, thus supporting the feasibility of constructing a large (>30,000 observations), standardised dataset pooled from various study protocols. These initial positive findings are encouraging to future researchers who may wish to administer TOPICS-MDS instrument within their own research protocols to further this initiative. The unique implementation strategy of TOPICS-MDS has several inherent strengths. First, integrating a standardised data collection tool into pre-existing research protocols is a highly efficient and cost-effective method to generate data on a large number of respondents. Moreover, by collecting uniform individual-level data, we counter traditional obstacles that impede meta-analysis, such as select reporting of aggregate data or differences in exposure/outcome operational definitions[25,26]. Lastly, with appropriate statistical considerations, the pooled data from TOPICS-MDS may be able to have broader generalizability than individual research studies[27]. 

Preliminary analyses of TOPICS-MDS revealed that a considerable proportion of recruited older persons experienced some form of disutility, whether related to morbidity, ADL or quality of life, thus alluding to the underlying extent of frailty. Defined as an increased vulnerability to adverse health outcomes following a stressor event[28], frailty can serve as a stronger indicator for geriatric intervention than chronological age[29]. For this reason, identifying frailty on a patient-level can result in more effective case management, and on a population-level, can lead to improved distribution of health services. Although there are several valid methods to measure frailty[28], the calculation of a frailty index based on the accumulation of deficits in health[30] (i.e. symptoms, morbidities and/or functional limitations) can be easily applied in large-scale population studies[31-33]. Thus, given the range of deficits captured within TOPICS-MDS, a frailty index can be derived to provide another important indicator of health in the database. 

TOPICS-MDS is not without limitations. Arguably, alternative metrics to those included may have permitted a more detailed investigation of outcomes of interest. However, TOPICS-MDS data collection instrument was designed to achieve a critical balance between content and succinctness. Moreover, although the data collection instrument was comprised of well established health scales, given the different sampling frameworks of individual research projects, further methodological investigations are necessary to assess if reliability, validity and generalizability are upheld in the overall sample population. Specifically, future studies examining cluster effects, heterogeneity and patterns in missingness are warranted to maximise the utility and interpretation of the data. 

Still, TOPICS-MDS should not only be seen as an endeavour to create a minimal dataset in older persons’ health and wellbeing but also as a large-scale data sharing initiative, which in itself is an important scientific output. Data sharing has the potential to provoke positive changes in public health strategies, improve project cost-effectiveness and enhance scientific integrity[34]. These advantages have become increasingly recognised throughout scientific communities, consequently prompting 17 major funders of public health research to draft a joint statement supporting public data repositories[34,35]. Nonetheless, while advances have been made in biomedical spheres, data sharing remains largely elusive in public health research[35]. Commonly cited barriers include data protection legislation, potential overlap in analyses and funding limitations[36]. Other underlying issues, such as self-perceived proprietorship over databases and the competitive demand to increase published output, also contribute to a research culture which is not conducive with data sharing[35,36]. Thus, despite the potential for increased citation rates[37] and journal policies advocating open access, the release of data is not always achieved in practice[38,39]. 

In light of these complications, TOPICS-MDS Project Group and Steering Committee sought to proactively address potential obstacles in order to encourage a culture of data sharing from the initial phases of the project. Firstly, to comply with data protection legislation, external users will only be permitted to access a fully anonymised database. To circumvent issues related to publication rights, a brief moratorium in the release of data is implemented to afford research consortium members the opportunity to publish without conflicting data requests. Following this period, all Project Group members acceded that they would have to submit a data request to perform any additional analyses not initiated during the moratorium. To further protect the interests of external users, TOPICS-MDS Societal Board was established as a safeguard against preferential release of data. Lastly, like many public health research projects, TOPICS-MDS received fixed funding. To promote the continuance of the project, funding calls are being actively sought by the Project Group and it is envisioned that TOPICS-MDS will be incorporated into future studies on older persons’ health. Opportunities to link data with permanently funded institutions are also being explored. 

Nonetheless, while these aforementioned measures are fundamental for data sharing, we believe that the strong commitment of all involved stakeholders underlies the success of this public data repository to date. Based on our experience, building TOPICS-MDS without collaborating with the researchers who collected the data would have been ineffective. Rather, we found keeping researchers engaged through regular updates and assistance with the data submission process were instrumental to the sustainability of the data sharing initiative. 

TOPICS-MDS will be open for external requests in the last quarter of 2013; full details on how to submit a request will be made available through TOPICS-MDS website at: http://www.topics-mds.eu. Additional background information, the TOPICS-MDS data dictionary and relevant syntaxes can also be accessed on the website. Documents are available in English and Dutch.

In conclusion, TOPICS-MDS represents a strong example of a public data repository with wide reaching potential. Understanding the health challenges experienced by older persons and informal caregivers can help inform the reconfiguration of contemporary care models to achieve a more integrated and proactive heath services system. Although based in the Netherlands, such findings are timely and relevant for many industrialised countries where increasing health-related expenditure has necessitated the evaluation of contemporary health care delivery.
